# Overexpression of BID in thyroids of transgenic mice increases sensitivity to iodine-induced autoimmune thyroiditis

**DOI:** 10.1186/1479-5876-12-180

**Published:** 2014-06-23

**Authors:** Su He Wang, Yongyi Fan, James R Baker

**Affiliations:** 1 Michigan Nanotechnology Institute for Medicine and Biological Sciences, Ann Arbor, Michigan, USA; 2 Department of Internal Medicine, University of Michigan, Ann Arbor, Michigan, USA

**Keywords:** BID, Iodine, Autoimmune thyroiditis, Mice, Transgene

## Abstract

**Background:**

BID functions as a bridge molecule between death-receptor and mitochondrial related apoptotic pathways to amplify apoptotic signaling. Our previous studies have demonstrated a substantial increase in BID expression in primary normal thyroid epithelia cells treated with inflammatory cytokines, including the combination of IFNγ and IL-1β or IFNγ and TNFα. The aim of this study was to determine whether an increase in BID expression in thyroid can induce autoimmune thyroiditis.

**Methods:**

A transgenic mouse line that expresses human BID in thyroid cells was established by fusing a mouse thyroglobulin (Tg) promoter upstream of human BID (Tg-BID). We tested whether the increased expression of pro-apoptotic BID in thyroid would induce autoimmune thyroiditis, both in the presence and absence of 0.3% iodine water.

**Results:**

Our data show that Tg-BID mice in a CBA/J (H-2 k) background do not spontaneously develop autoimmune thyroiditis for over a year. However, upon ingestion of iodine in the drinking water, autoimmune thyroiditis does develop in Tg-BID transgenic mice, as shown by a significant increase in anti-Tg antibody and mononuclear cell infiltration in the thyroid glands in 30% of mice tested. Serum T4 levels, however, were similar between iodine-treated Tg-BID transgenic mice and the wild type mice.

**Conclusions:**

Our data demonstrate that increased thyroid expression of BID facilitates the development of autoimmune thyroiditis induced by iodine uptake. However, the overexpression of BID itself is not sufficient to initiate thyroiditis in CBA/J (H-2 k) mice.

## Background

Autoimmune (Hashimoto’s) thyroiditis is characterized by lymphocytic infiltration of the parenchyma, causing a dense accumulation of lymphocytes, plasma cells, and macrophages with germinal center formation and thyroid enlargement. Although the pathogenesis of autoimmune thyroiditis is not entirely clear, studies have demonstrated that increasing the local production of inflammatory cytokines in the thyroid microenvironment plays a critical role in facilitating apoptosis in thyrocytes, leading to autoimmune thyroiditis [1-3]. Among various inflammatory cytokines, IFN-γ, TNF-α and IL-1β appear to be critical since the combination of two such cytokines can significantly facilitate the development of experimental autoimmune thyroiditis (EAT) in mice [1,3,4], a well-recognized animal model of autoimmune thyroiditis.

BH3 interacting-domain death agonist (BID) is a pro-apoptotic Bcl-2 family member that functions as a bridge molecule between two classic apoptotic pathways to augment apoptotic signaling. Our previous studies showed that the expression of BID in primary normal thyroid cells was significantly increased by inflammatory cytokines in vitro [3]. The pretreatment of those inflammatory cytokines can also sensitize thyroid epithelia cells to death-receptor mediated apoptosis [2,3]. This finding suggests a potential role for BID in the pathogenesis of autoimmune thyroiditis. A number of recent reports have also indicated the involvement of BID in the development of other autoimmune diseases [5-7]. We therefore hypothesized that the overexpression of BID plays a positive role in the development of autoimmune thyroiditis. To test this hypothesis we first established a transgenic mouse line that expresses human BID specifically in the thyroid, and then tested whether the overexpression of BID alone is sufficient for the development of autoimmune thyroiditis.

It has also been shown that there is connection between iodine ingestion and autoimmune thyroiditis. Indeed, iodine administration has been implicated in increasing incidence and/or severity of spontaneous thyroiditis in BB/W rats, CS chickens, hamsters and NOD-H-2 h4 mice [8]. CBA/J (H-2 k) mice, however, are genetically susceptible to EAT induced by Tg but are resistant to EAT induced by iodine intake alone [8-10]. In this study, we employed CBA/J (H-2 k) mice to examine whether the overexpression of BID can overcome this resistance, and sensitize these mice to iodine-induced autoimmune thyroiditis.

## Methods

### Tg-BID transgenic mice

To produce a transgenic mouse line that overexpresses human BID specifically in the thyroid, a rat thyroglobulin (Tg) promoter was fused with the human BID gene. To accomplish this, the rat Tg promoter region was amplified by PCR using rat genomic DNA as a template and primers with internal Afl II/KpnI sites (Forward primer 5′ata tac tta ctt aag ctg cag aca agc agg cat gc-3′; Reverse primer 5′tta act ata ggt acc tac tca aat gat ggg gta gga g-3′). After digestion, the fragment was fused upstream of 0.9 kb of BID cDNA sequence (accession no.AF042083). Then, Tg-BID transgene was cloned into pCMV or pcDNA3.1 (Invitrogen). After confirming the correct sequence for the Tg promoter and insertion site, a 2515 bp Tg-BID transgene fragment released by Bg1II/NaeI was used to develop transgenic mice in the Transgenic Core of our institute. The Tg-BID transgene was microinjected into eggs from C57BL/6 J x CBA/J (H-2 k) female mice. The Tg-BID positive C57BL/6 J x CBA/J (H-2 k) mice were confirmed by PCR. CBA/J (H-2 k) mice, which have a genetic predisposition to develop EAT [8,9], were crossed with the Tg-BID positive C57BL/6 J x CBA/J (H-2 k) mice to produce C1 mice. Subsequent generations (C2-C8) were produced with similar backcrosses. All animal experiments were conducted following a protocol approved by the University of Michigan Committee on the Use and Care of Animals (UCUCA) (Approval Ref No. PRO00005076).

### RNA isolation and RT-PCR

RNA was extracted from mouse thyroid tissues by Trizol (Invitrogen) and converted to cDNA by reverse transcription, using M-MLV Reverse Transcriptase. The cDNA was then amplified by real time PCR, using the Mastercycler® ep real plex (Eppendorf). The forward and backward primers for BID were 5′-*AAGAAGGTGGCCAGTCACAC*-3′ and 5′-*GTCCATCCCATTTCTGGCTA*-3′, respectively. The β-actin (5′-*ACTGCTCTGGCTCCTAGCAC*-3′ and 5′-*ACATCTGCTGGAAGGTGGAC*-3) was also measured by RT-PCR from the same RNA samples and was used as an internal control. mRNA expression was quantified using the comparative CT (cross threshold, the PCR cycle number that crosses the signal threshold) method as previously described [4].

### Protein isolation and detection

Mouse tissue protein was isolated from Trizol homogenized tissues after extraction of RNA, according to the manufacturer’s protocol (Invitrogen). Western blots were performed according to the previous procedures [1-4]. BID expression was also detected by immunohistochemistry. Briefly, mouse primary thyroid cells cultured in 8-well plates were stained with mouse anti-human BID antibody (IgG1, BD Transduction Laboratories), then stained with goat anti-mouse IgG1-Cy2 and DAPI.

### Induction and evaluation of autoimmune thyroiditis

8-week old, female BID transgenic mice and age matched wild type mice were given iodine water (0.3% of Sodium iodide) for 8 weeks. Evaluation of thyroiditis was done by measuring serum anti-Tg antibody and examining thyroid histopathology in BID transgenic mice and wild type mice. 10 mice were included for each group. Serum anti-Tg antibody was determined by solid-phase ELISA as previous described [11]. Briefly, plastic microtiter plates were coated with 100 μl of murine Tg (10 μg/ml), and sera from individual mice in 1:50, 1:100, and 1:200 dilutions were added to each well. An alkaline phosphatase-conjugated, sheep anti-mouse IgG (Sigma) was added to determine anti-Tg IgG. Thyroid histopathology was examined as described in our previous publications [1,4].

### Statistical analysis

The values given are presented as mean ± SD. Statistical analysis was performed using one-way analysis of variance (ANOVA) followed by Student’s t test. p < 0.05 was considered as significant.

## Results

### Establishment of transgenic mice with BID overexpression in thyroid gland

**Figure 1:**
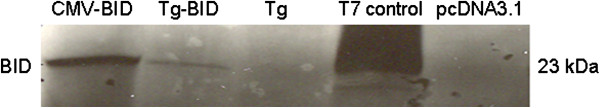
**Expression of Tg-BID plasmid in the TNT T7 Quick Coupled transcription/translation system.** 1 μg of CMV-BID, Tg-BID, Tg, and pCDNA3.1 were added to TNT T7 quick reaction. A 2.5-μl aliquot of each reaction was separated on a 15% SDS-PAGE gel and BID protein was recognized by a specific anti-BID antibody.

**Figure 2:**
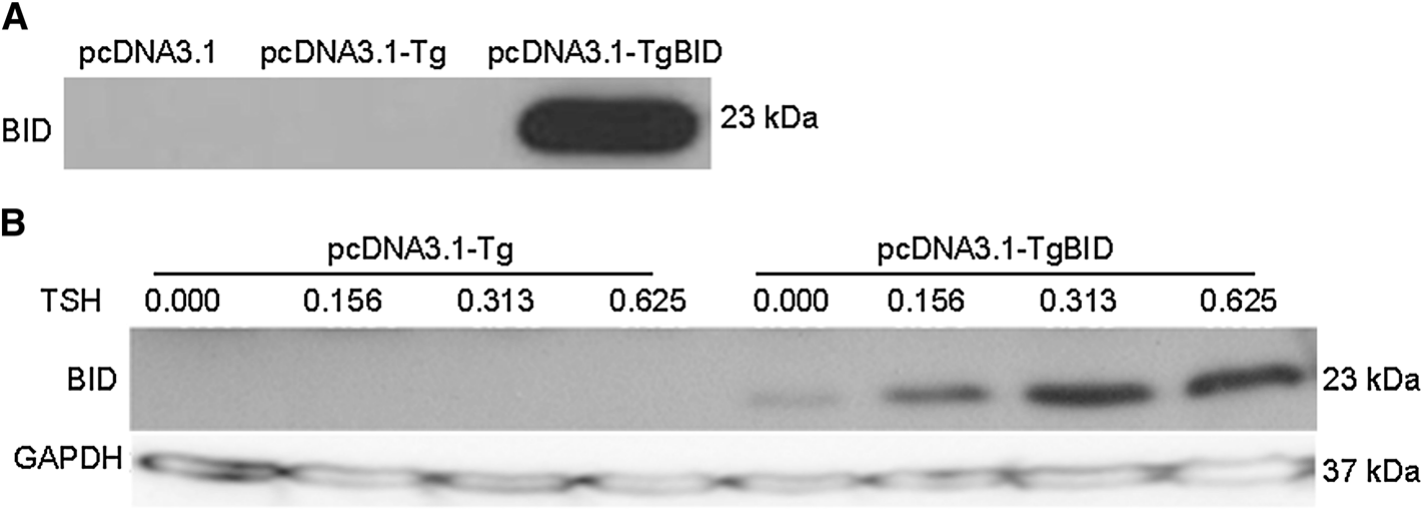
**Transgene BID protein expressed in FRTL-5 cells and FRTL-5 cells treated with TSH.** Total protein was isolated from FRTL-5 cells transfected with pcDNA3.1-Tg-BID and subjected to Western blot for the transgene Bid protein **(A)**. Total protein from FRTL-5 cells transfected with pcDNA3.1-TgBID and treated with a series of diluted TSH for 72 hours was also isolated and subjected to Western blot to detect BID **(B)**. GAPDH was used as a loading control.

To generate Tg-BID transgenic mice, the rat Tg promoter region was fused upstream of the Bid transgene and cloned into pCMV to form CMV-BID. Sequence analysis was used to confirm that the Tg-BID plasmid had the correct sequence for the Tg promoter and insertion site. To verify expression of BID from the Tg-BID construct, the TNT-coupled transcription/translation system was used according to the manufacturer’s protocol. Under *in vitro* transcription/translation conditions, the Tg-BID construct expressed a BID protein of the predicted size (23 kD), which was the same size produced by the CMV-BID (Figure 1). It is obvious that CMV promoter is much stronger than the Tg promoter. This verified that the BID protein translated from these transgenes can be recognized by anti-BID antibody.The expression of the transgene has also been validated in transfected FRTL-5 cells. The pcDNA3.1-Tg-BID transgene was efficiently expressed in FRTL-5 cells transfected with pcDNA3.1-Tg-BID, but not in FRTL-5 cells transfected with control pcDNA3.1-Tg plasmid (Figure 2A). In a second set of experiments, FRTL-5 cells transfected with pcDNA3.1-Tg-BID were cultured in complete media without TSH for one week, followed by the addition of 0–0.625 mU/ml of TSH for 72 hours. It is known that TSH is able to activate Tg promoter. Figure 2B shows that BID protein expression was regulated by TSH, confirming the functionality of the Tg promoter.

### Assessment of spontaneous thyroiditis in Tg-BID CBA/J (H-2 k) mice

**Figure 3:**
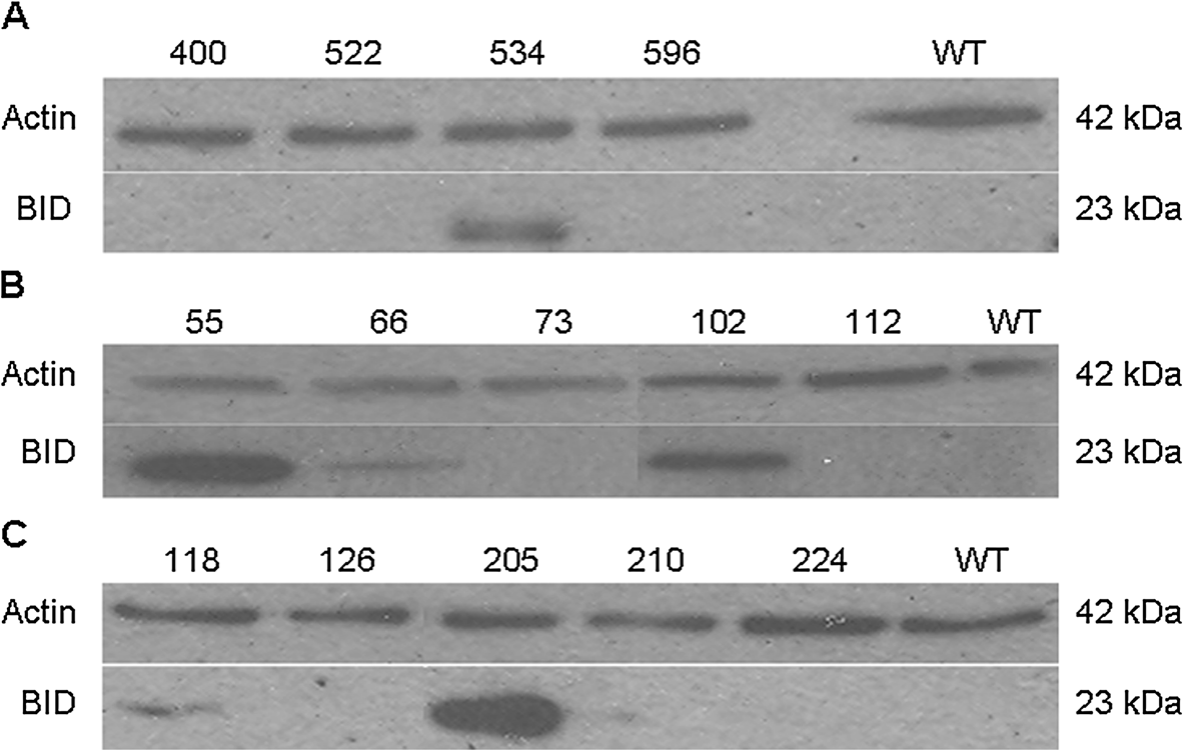
**Expression of transgene Tg-BID in the thyroid of the founder (A), C1 (B) and C2 (C) mice.** Mouse thyroid cells were isolated and cell lysates prepared and subjected to Western blot for BID. Actin was detected as a control for equal loading. The number represents different clones of transgenic mice. WT: wild type.

**Figure 4:**
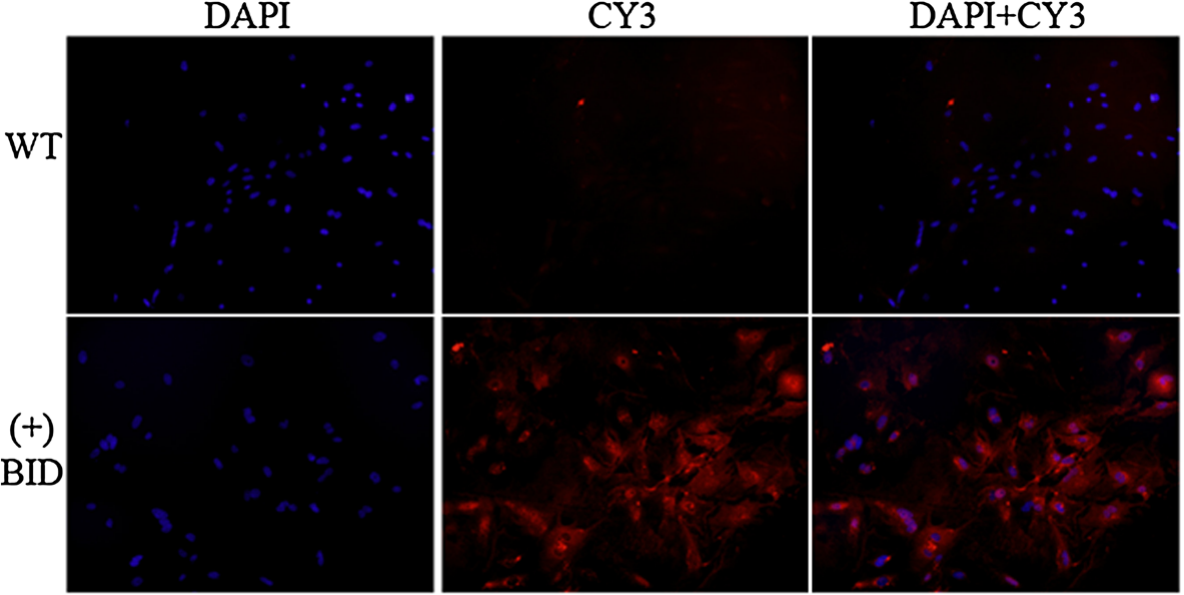
**Immunofluorescent staining of cultured primary mouse thyrocytes from wild type and Tg-BID postive transgenic mice.** Left panel: DAPI nuclear staining of wild type (WT) and transgene positive cells. Middle panel: CY3-BID staining of WT and transgene positive cells. Right panel: Merged DAPI and CY3-BID staining of WT and transgene positive cells.

Tg-BID positive C57BL/6 J X CBA/J (H-2 k) mice were crossed with CBA/J (H-2 k) mice, which are a strain genetically susceptible to EAT [8-10], to produce C1 mice, and subsequent generations were made with similar backcrosses. RNAs extracted from the C4 transgenic mice thyroids demonstrated message for Tg-BID positive mice, but not in DNA negative mice (Table 1), nor in other tissues in the transgene positive mice (data not shown). The expression of BID protein was examined in the thyroid from the founder to C4 transgenic mice using Western blot and found that a considerable number of mice expressed BID (Figure 3). Mice with high, medium and low levels of Tg-BID were defined based on the relative level of Tg-BID protein as compared to the actin control. Clones with very high levels of Tg-BID relative to actin controls were considered “high” (e.g., clones 55 and 205 in Figure 3); clones with Tg-BID levels that were similar to the actin control were considered “medium” (e.g., clones 534 and 102); and clones with very low levels of Tg-BID relative to the control were considered “low” (e.g., clones 66 and 118). Clones positive for BID by Western blot were confirmed by immunofluorescent staining of cultured primary mouse thyrocytes (Figure 4). It was noted that the levels of BID protein in the positive clones were various, which enabled us to divide the transgenic mice into high, medium and low Tg-BID expression group for the further function study.

**Table 1:** Quantification of Tg-BID mRNA expression in mouse thyroid by RT-PCR

**Mice**	**Subgroup**	**n**	**Tg-BID level**
Wild type		8	0
Transgenic	Low	8	5
Transgenic	Medium	8	37
Transgenic	High	8	118

Though the levels of Tg-BID proteins were highly variable in those transgenic mice, no anti-Tg antibody was detectable in sera. In addition, serum T4 levels were not different between Tg-BID transgenic mice and wild-type mice. Furthermore, the gross appearance and microstructure of thyroid glands in all transgenic mice appeared similar to that of the wild-type mice, suggesting that the overexpression of BID itself has no impact on thyroid function in terms of T4, and does not cause a notable autoimmune response in the thyroid gland.

### Iodine induced autoimmune thyroiditis in Tg-BID transgenic CBA/J (H-2 k) mice

**Figure 5:**
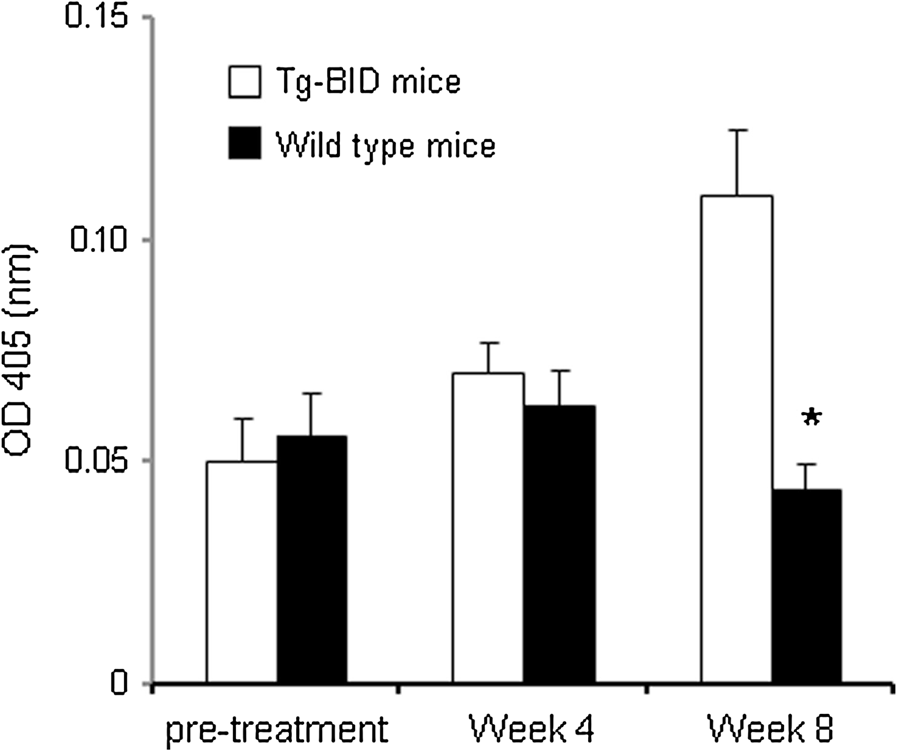
**Elevation of serum thyroid autoantibody anti-Tg in Tg-BID transgenic CBA/J mice treated with iodine.** Sera were collected from the mice before iodine administration (pre-treatment), 4 weeks after iodine administration and 8 weeks after iodine administration. Anti-Tg antibody was measured by solid-phase ELISA and results were expressed as OD 405 ± SD of 1:200 dilution of serum from ten individual mice per group.

**Figure 6:**
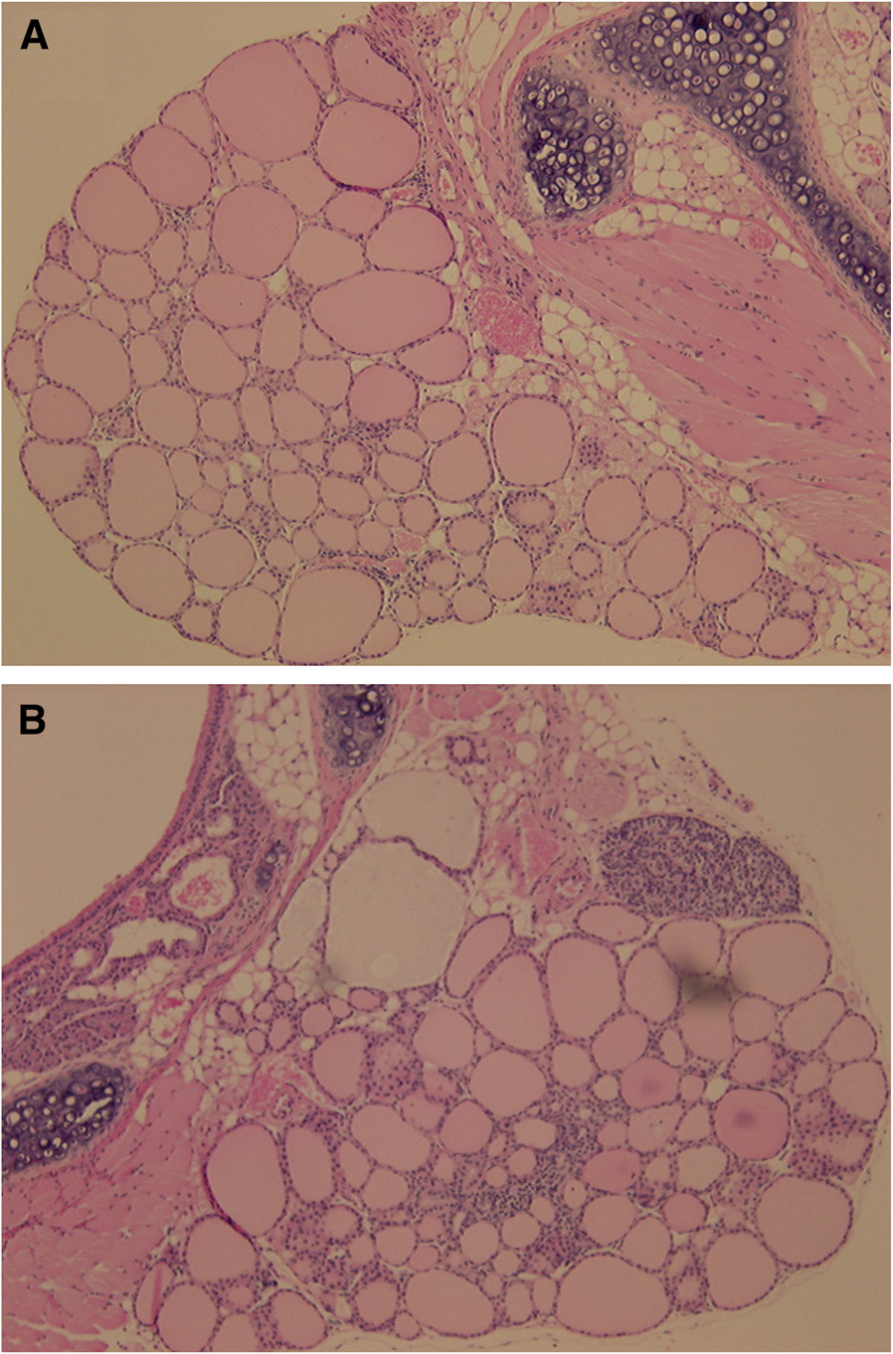
**Thyroid histology.** Representative hematoxylin and eosin staining of thyroid glands of the wild type CBA/J (H-2 k) mice **(A)** and Tg-BID transgenic CBA/J (H-2 k) **(B)**. Obvious inflammatory infiltration was observed in the latter but not in the former. Magnification =20X.

As demonstrated above, no spontaneous thyroiditis was observed in Tg-BID transgenic CBA/J (H-2 k) mice. To investigate whether BID can cooperate with other thyroiditis risk factors to facilitate the development of autoimmune thyroiditis, we treated mice with iodine, a known risk factor for thyroiditis [10,12,13]. Mice treated with 0.3% iodine begin to show an increase in anti-Tg antibody at Week 4 in Tg-BID transgenic mice compared with wild type CBA/J (H-2 k) mice, and the level of anti-Tg antibody was significantly higher at Week 8 (* p < 0.01, Figure 5). Consistent with this increase in anti-Tg antibody, 3 of 10 Tg-BID transgenic CBA/J (H-2 k) mice showed thyroid gland mononuclear cell infiltration whereas no thyroids of the wild type CBA/J (H-2 k) had this phenotype (Figure 6). Despite the increase of serum anti-Tg antibody and mononuclear cell infiltration, serum T4 levels were similar between iodine-treated Tg-BID transgenic CBA/J (H-2 k) mice and wild-type CBA/J (H-2 k) mice. These findings suggest that the thyroid function of the Tg-BID transgenic mice appears within the normal range after receiving 8-week iodine in the drinking water, even though iodine uptake does result in a certain degree of autoimmune responses such as autoantibody production and mononuclear cell infiltration in Tg-BID transgenic mice.

## Discussion

Our previous work demonstrated that the Fas pathway in thyrocytes can be activated by Fas ligand in the presence of combinations of pro-inflammatory cytokines such as IL-1β, IFNγ and TNFα [1,2]. Further experiments revealed that the induction of apoptosis in thyrocytes is correlated with an increase of BID and Bak levels but a decrease of p44/42 mitogen-activated protein kinase activity [2,14,15]. These findings suggest that the Fas apoptotic pathway activity may be further amplified by the bridge pro-apoptotic molecule BID, leading to the release of Bak from the mitochondria and activation of caspases. Thus, apoptosis of thyrocytes in EAT or autoimmune thyroiditis is dependent on both cell death receptors and mitochondrial elements, in which BID plays a critical role [16,17]. In contrast to most apoptotic activity, apoptotic cell death in EAT or autoimmune thyroiditis is not anti-inflammatory [18]. Instead, apoptotic thyroid cells and secondary necrotic cells induce a pro-inflammatory environment, which may provide sufficient levels of self-antigens to intensify a deregulated immune response [18,19]. The occurrence of inflammation is now known to be closely associated with a BID-related pathway that is either dependent or independent of apoptosis [20-22].

To test whether the genetic changes in BID play a role in the development of autoimmune thyroiditis, we successfully developed a transgenic mouse line in which BID is specifically over-expressed in the thyroid. We observed Tg-BID transgenic mice for 15 months, and during this period these mice remained healthy. These mice showed no obvious difference in activity and appearance compared with the control mice. In addition, no difference in thyroid histology and morphology were observed. We found that regardless of the level of BID in the thyroid of these transgenic animals, no differences were observed in the growth of mice, their activity, or serum T4 levels. Importantly, the classical features of thyroiditis, such as thyroid auto-antibodies, did not appear in any of the transgenic Tg-BID mice. These findings indicate that the overexpression of BID alone is not sufficient to cause the pathologic phenotype characteristic of spontaneous thyroiditis in mice.

The observation that Tg-BID transgenic mice do not develop spontaneous autoimmune thyroiditis is not totally unexpected. Autoimmune thyroiditis is a disorder with multiple causative factors including genetic, environmental, and immunological elements [18,23]. Among these elements, iodine is able to exert pleiotropic effects on either metabolic or immunological processes of thyroid. While iodine is an indispensable constituent of the two major thyroid hormones T3 and T4 [24], it contributes to the development of autoimmune thyroiditis by enhancing the antigenicity of thyroglobulin and reducing regulatory T cells [13,25]. Though iodine is known to contribute to the development of autoimmune thyroiditis [8-10], it may not be able to do so without other co-factors. For example, iodine is able to induce thyroiditis in nearly 100% of NOD.H-2 h4 mice that express I-Ak on the NOD genetic background, but cause none in mice such as CBA/J (H-2 k) (H-2 k) and NOD.SWR(H-2(q)) that do not carry I-Ak [8]. It is unknown whether mice with BID overexpression would sensitize thyroiditis induced by iodine. To this end, we employed iodine-resistant CBA/J (H-2 k) mice in this study. We show that upon iodine administration, Tg-BID transgenic CBA/J (H-2 k) mice do indeed develop autoimmune thyroiditis in about 8 weeks, which is evident by a significant increase in serum anti-Tg autoantibody and 30% of Tg-BID transgenic CBA/J (H-2 k) mice having mononuclear cell infiltration into the thyroid glands. The timing of iodine-induced autoimmune thyroiditis in Tg-BID transgenic CBA/J (H-2 k) mice appears to be quite similar to that in NOD-H-2 h4 mice [8].

## Conclusions

Our study has demonstrated that the increasing BID expression specifically in thyroid does not cause autoimmune thyroiditis. However, mice with thyroid-specific BID overexpression are at high risk of development of autoimmune thyroiditis induced by known pathogenic factors such as iodine. These findings support the common concept that autoimmune thyroiditis is a multi-factorial disease, resulting from interplay of genetic, environmental, and endogenous factors [18,23].

## Abbreviations

BID: BH3 interacting-domain death agonist; EAT: Experimental autoimmune thyroiditis; Tg: Thyroglobulin.

## Competing interest

All authors have no conflict of interest.

## Authors’ contributions

SHW and JRB designed the study, and analyzed results. SHW and YF carried out experiments and drafted the manuscript. All authors have read and approved the final manuscript.
